# The Rise of Pregnancy Apps and the Implications for Culturally and Linguistically Diverse Women: Narrative Review

**DOI:** 10.2196/mhealth.9119

**Published:** 2018-11-16

**Authors:** Jo-anne Patricia Hughson, J Oliver Daly, Robyn Woodward-Kron, John Hajek, David Story

**Affiliations:** 1 Research Unit for Multilingualism and Cross-Cultural Communication University of Melbourne Parkville Australia; 2 Western Health Sunshine Hospital St Albans Australia; 3 Department of Medical Education University of Melbourne Parkville Australia; 4 Anaesthesia, Perioperative and Pain Medicine Unit Melbourne Medical School University of Melbourne Parkville Australia

**Keywords:** culture, emigrants and immigrants, health communication, information-seeking behavior, literacy, maternal health, mHealth, mobile phone, pregnancy, self-care, vulnerable populations

## Abstract

**Background:**

Pregnancy apps are a booming global industry, with most pregnant women in high-income countries now using them. From the perspective of health care and health information provision, this is both encouraging and unsettling; the demand indicates a clear direction for the development of future resources, but it also underscores the importance of processes ensuring access, reliability, and quality control.

**Objective:**

This review provides an overview of current literature on pregnancy apps and aims at describing (1) the ways in which apps are used by women, in general, and by those of a culturally and linguistically diverse (CALD) background; (2) the utility and quality of information provided; and (3) areas where more research, development, and oversight are needed.

**Methods:**

We chose a narrative review methodology for the study and performed a structured literature search including studies published between 2012 and 2017. Searches were performed using MEDLINE, EMBASE, and CINAHL databases. Studies were identified for inclusion using two separate search criteria and strategies: (1) studies on pregnancy apps and pregnant women’s use of these apps and (2) studies on CALD pregnant women and their use of technology for accessing information on and services for pregnancy. Overall, we selected 38 studies.

**Results:**

We found that pregnancy apps were principally used to access pregnancy health and fetal development information. Data storage capability, Web-based features or personalized tools, and social media features were also popular app features sought by women. Lower rates of the pregnancy app uptake were indicated among lower-income and non-English-speaking women. Preliminary evidence indicates that a combination of technological, health literacy, and language issues may result in lower uptake of pregnancy apps by these groups; however, further investigation is required. A marked limitation of the health app industry is lack of regulation in a commercially dominated field, making it difficult for users to assess the reliability of the information being presented. Health professionals and users alike indicate that given the choice, they would prefer using pregnancy apps that are relevant to their local health care context and come from a trusted source. Evidence indicates a need for greater health professional and institutional engagement in the app development, as well as awareness of and guidance for women’s use of these resources.

**Conclusions:**

This is the first review of pregnancy app use, types of information provided, and features preferred by pregnant women in general and by those of a CALD background in particular. It indicates the demand for access to accurate information that is relevant to users, their community, and their associated health services. Given the popularity of pregnancy apps, such apps have enormous potential to be used for the provision of accurate, evidence-based health information.

## Introduction

Engaging with pregnancy apps appears to have become a routine part of the maternity experience for many expectant mothers. Globally, there are more pregnancy apps than for any other medical topic [1], which attests to their ever-increasing popularity. Internationally, the majority of smartphone owners are women [2], with indications that pregnancy itself is an incentive to buy a smartphone [3]. In advanced economies, like the United States and Australia, between 72% and 89% of the general population, and 92%-95% of 18- to 34-year olds, owned smartphones in 2015 [4,5].

There are several reasons for the high uptake of apps by women. Pregnancy is a normal life process influenced by many social and cultural practices and interactions with the health system. The types of information and resources sought by women during pregnancy span many domains such as health care, social, cultural, and material. Accordingly, pregnancy apps incorporate a range of platforms with diverse features catering to these diverse domains. We propose that the range of pregnancy apps can be categorized along a continuum from entertainment at one end through to health care at the other. Common features of pregnancy apps include the following: information provision, pregnancy tracking, record keeping, and gestation calculation. Of the plethora of pregnancy apps designed for entertainment purposes [6], games reportedly constitute the highest number of pregnancy apps [7], alongside Web-based shopping for pregnancy-related products, gender predictors, and baby name generators [8]. In addition, there are apps that are not pregnancy specific, such as for social networking, which may take on a specialized function during a woman’s pregnancy, providing an important link to social connection and support [9,10]. Some apps offer multifunctionality while others are dedicated to a single function. While some apps clearly belong to either end of the continuum, many apps blend multiple functions that could place them at both ends of the same continuum.

While there is a growing popularity of pregnancy apps, it is unclear to what extent they actually address the needs of particular groups of women, such as culturally and linguistically diverse (CALD) populations. In this review, the term CALD refers to people living in an English language-dominant context who are of non-English-speaking background (NESB) and who may be bi-, tri-, or multilingual, but generally have low proficiency in English. CALD groups require particular consideration, given cultural variabilities in maternity practices of different ethnic groups [11], lower levels of literacy [12], health literacy [13], health care access [14], and concomitant poorer perinatal outcomes of some CALD groups in comparison to non-CALD women [15]. Thus, it is crucial to ascertain whether digital technologies improve outcomes for CALD and other disadvantaged groups or simply perpetuate the *status quo* [16].

This review provides an overview of women’s preferences for and use of pregnancy apps as resources for pregnancy information, the effectiveness and acceptability of such resources, and their shortcomings, including unregulated development that may affect the quality of information provided. Based on the findings, recommendations are made regarding future research and features that need to be included in pregnancy apps to ensure that they provide useful, reliable information in a format that is accessible to the general population, including CALD users.

## Methods

### Research Question

The research question guiding this narrative review initially focused on studies examining the pregnancy app usage in CALD women. The review was motivated by the authors’ involvement in providing digital health information to CALD pregnant women, in particular via smartphones. However, the scoping searches revealed a gap in the literature, prompting a broader review of women’s use and uptake of pregnancy apps more generally, including issues related to their design and content.

### Search Strategy and Selection of Studies

A structured literature search was performed including studies published between 2012 and 2017. Searches were carried out using MEDLINE, EMBASE, and CINAHL databases. Given the varied nature of the publications under review, studies were identified for inclusion using two search strategies: (1) studies of pregnancy apps and pregnant women’s use of these apps and (2) studies of CALD pregnant women and their use of technology for accessing information on and services for pregnancy. A sample search strategy used is reproduced in Multimedia Appendix 1.

### Inclusion and Exclusion Criteria

For studies reporting women’s use of pregnancy apps, type 1 studies, the ones dedicated to a particular health topic in pregnancy (eg, nutrition education and breastfeeding), were excluded, although studies reporting on the general app usage to inform the design of pregnancy topic-specific resources were deemed suitable for inclusion. Studies of apps designed for use in clinical contexts and requiring the coparticipation of health professionals were also excluded unless they contained explicit content regarding CALD usage. These delimitations were set because (1) the authors were searching for information on designing an app on pregnancy, rather than an app on a specific pregnancy-related topic, and (2) the app was for pregnant women to use themselves, rather than an app for use with or by health professionals.

**Figure 1:**
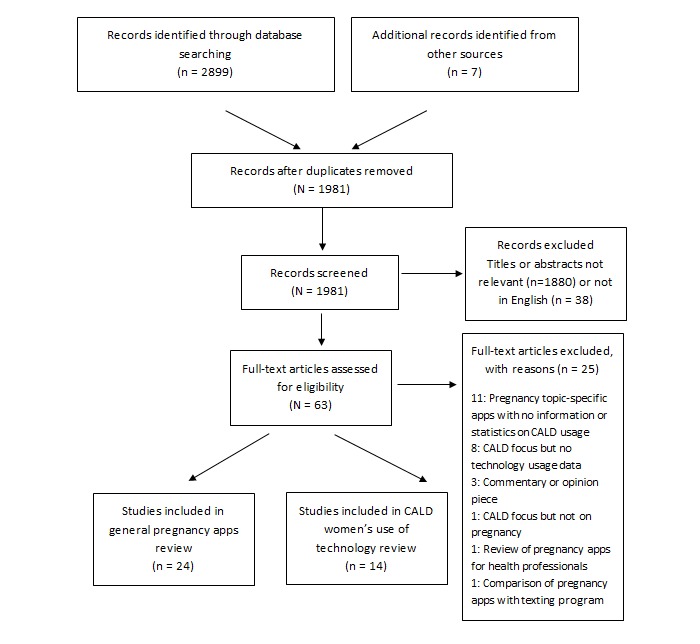
Literature search. CALD: culturally and linguistically diverse.

In the absence of substantial material on CALD women’s use of apps, but seeking to gain an appreciation of factors that may help inform CALD usage of pregnancy apps, we additionally searched for studies that included information about CALD use of technology in pregnancy more broadly (including mobile phone usage, internet usage, general app or smartphone use). No exclusion criteria were applied to these studies provided they gave detailed information about the CALD women’s usage and preferences with regard to digital technology. Further relevant articles were identified through manual searches of reference lists. In total, 38 studies were selected for inclusion in the review (Figure 1).

### Study Analysis

Once accepted for inclusion, type 1 studies (n=24) were analyzed for any content pertaining to the pregnancy app usage, preferences in app features or design, or issues associated with app usage and type 2 studies (n=14) were analyzed for content concerning their attitudes toward and uptake or acceptance of digital technologies related to pregnancy care. These results were tabulated and are reproduced in Multimedia Appendices 2 and 3.

## Results

### How Pregnant Women Use Apps

#### The General Population

Information seeking is a key motivation for women using pregnancy apps. Certainly, the largest category of apps available on the market involves those providing information about pregnancy [1]. We found limited research providing evidence of the exponential rise in popularity of apps for accessing pregnancy information. A 2013 Australian study found that 40% of 35 participants had used at least one smartphone app to access pregnancy information [17]. In 2016, a larger Australian survey study of 410 women reported the proportion of pregnancy app users as 73%, the vast majority (92%) finding them to be useful [8,18]. The most valued feature was providing useful information (83%), mainly about fetal development (89%) or pregnancy-related changes in their bodies (71%) [8]. In a Korean study [19] evaluating app use among 193 pregnant women, most participants used apps to obtain information, and the most frequently sought information was on risks and diseases during pregnancy (17%), physical changes related to a normal pregnancy (16%), prenatal education (15%), and breastfeeding and baby food (13%). An analysis of search engine use demonstrated pregnancy information seeking to be a primary function of women’s digital pregnancy-related activities in several studies [6,19,20]. One US study found that Google searches were the most common mode of information seeking, particularly early in pregnancy [20]. An important factor for many women was the ability to obtain information instantly and easily [6,19,21,22].

The most popular apps have Web-based features [1]. When considering pregnancy apps, Web-based and mobile features include regular notifications, videos, and data storage capability, for example, for photos; taking notes; pregnancy tracking; and “personalized tools to assess nutrition, fitness, and weight” [18,23]. In addition, women value pregnancy apps and digital platforms “that are multifunctional and interact with each other” [6,24]. Participants in a recent German study strongly advocated for apps that had the functionality to provide individualized feedback, “communication platforms” for pregnant women and medical professionals, and indicated a desire for the integration of apps into routine clinical and pregnancy care [22]. Pregnant women interviewed to inform the design of a Dutch pregnancy app wanted all of the abovementioned features as well as a push notification system to remind them of tasks they needed to accomplish, an alert system to tell them if any monitored signs became irregular or dangerous, and a social media module for connecting with other pregnant women [25].

Social media use (eg, use of Facebook, discussion forums) has frequently featured in research on pregnancy information seeking and can be an important way for women to find support, develop networks, and foster emotional well-being [26,27]. Social media platforms, which are usually accessed via an app on women’s phones, also provide women with a setting in which they can acquire and share (nonmedical) knowledge and expertise about motherhood and are a helpful way for women to get an idea of whether their symptoms and experiences during pregnancy are “normal” [21]. Apps with social media functionality may be particularly useful in late pregnancy and the postnatal period when needs change from seeking information to seeking support, being part of a community, and avoiding isolation [26]. One small US study reported 82% of pregnant women used Web-based social networking sites, at least, once a day [20], and discussion forums and social media were the most used websites in a large-scale Irish study [28]. However, the appeal of social media is not universal, with one study reporting low levels of interest by lower-income American women in using social networking tools to discuss their pregnancy—they preferred to seek support from their existing social circles [29]. It should be noted that some of the references above are general studies on social media use that did not disclose how women accessed social media, for example, via apps on their phones or other interfaces, and are, therefore, not included in the summary tables (Multimedia Appendices 2 and 3).

#### Pregnancy App and Other Technology Use Among Culturally and Linguistically Diverse and Socially Disadvantaged Women

This section discusses results of studies reporting CALD (or ethnic or racial minority) groups’ use of technology during pregnancy and discusses whether or not, or to what degree, digital resources are acceptable and usable for these groups. We found only one study focusing on the pregnancy app use by CALD women [30], and most studies excluded women who were not proficient speakers of the given society’s dominant language, for example, English, in English-speaking countries. Yet, CALD communities make up a proportion of the population in most countries. For example, in the Australian setting, almost one-quarter of women giving birth in 2013 were born outside Australia in non-English-speaking countries [31]. There appears to be little provision of multilingual apps or apps for use with migrants, with only 2 multilingual apps found for use in maternity care as part of our literature search; both were developed in Europe for health professional-patient interactions [32,33]. While these are much-needed resources, they fulfill a different role to the type of resources examined by this review.

There is evidence that CALD women use technology to source pregnancy information. An Australian study examining sources of information used by women in pregnancy did include CALD-background respondents, with more than one-third of respondents listing English as a second or other language [34]; it should be noted, however, that participants’ English proficiency was sufficiently high to complete a written questionnaire in English. The study found that the internet was the most used resource by the NESB women in the study when seeking pregnancy-related information, although discussion with a midwife was deemed the most useful source of information [34]. While not reporting in any systematic way on app use, the study’s findings indicate that CALD women are receptive and *au fait* with current technology. Evidence in favor of the acceptability and effectiveness of technology for increasing the knowledge in CALD groups has also been provided in a study testing an English language Web-based intervention in an Australian health service, to increase the knowledge of gestational diabetes mellitus in a multiethnic and low health literacy population [35]. Participants’ knowledge scores increased across 3 domains from the pretest to the posttest. In the US context, a number of studies have indicated high ownership of smartphones and mobile phones in ethnic or racial minority groups and a preference for accessing Web-based content using these devices [36-39].

Nevertheless, others make the case that socially disadvantaged persons—including CALD people, especially those who have low English proficiency [40]—are further disadvantaged by digitization of health [16]. Some research suggests that pregnancy app engagement may be lower in areas of higher social disadvantage owing to factors such as less access to the internet and smartphones [41], as well as cultural and language barriers to app referral and content [42]. The existing research is inconclusive regarding whether, or to what extent, factors related to social disadvantage influence pregnancy app uptake, use, and utility. For instance, in the Australian study cited above [34], while NESB women mostly used the internet to source pregnancy information, it was also found that the internet use was significantly higher among those who had tertiary education. Conversely, a Dublin study informed by survey data from a large representative sample of pregnant women (n=522) collected in 2012-13, found that there was extremely high general internet usage *across all social strata* (97%), high smartphone ownership (61%), and widespread usage of pregnancy apps among smartphone owners (48%) [28]. A limitation of this study is that it does not provide information about CALD or literacy status, and it must be assumed that respondents had sufficient literacy skills and English proficiency to fill out a written survey in English. Similarly, a 2015 US study examining the willingness of pregnant women to engage with computer- or mobile phone-delivered weight-loss interventions reported a high general use of and access to the internet via a computer or mobile phone, although there was slightly less access among nonwhite women and women who already had children [43]. Around half of the 100 women surveyed (which comprised 61% white, 26% black, 6% Hispanic, and 7% Asian women) were willing to participate in a mobile-based internet intervention (49%), while 83% were willing to participate in a computer-based intervention. This study’s findings indicate that physical access to technology, then, may not be a significant limiting factor for marginalized groups in and of itself.

However, a person’s ability to successfully engage with and retrieve required information from a resource may be a strong barrier. Baum et al [16] have demonstrated how fundamental literacy and health literacy issues can interact with digital literacy to exclude already marginalized groups from successfully taking up digital technologies, creating a “vicious cycle of disadvantage”. The findings of Kraschnewski et al in their focus group study also support this contention [20]. The women in the study were of low socioeconomic status and all had smartphones and internet access; however, the women reported barriers to technology use, such as needing to conduct multiple searches to find the information they were seeking, and having concerns about receiving inaccurate information. It was suggested that these limitations might stem from a lower capacity to critically assess the source and accuracy of information. The authors expressed concern that the benefits of the internet and app-based tools may, thus, be limited by low electronic health (eHealth) literacy [20]. Low health literacy is proffered as a possible explanation for the presence of information “gaps” in women’s knowledge in another study of predominantly white women, from a range of educational and income levels, who demonstrated active information-seeking behavior during pregnancy using both Web-based and other strategies [44]. Another study of low-income, predominantly black or Hispanic, American pregnant women and mothers of young children found low use of digital health management practices among almost three-quarters of their 92 study participants [45]. While around half of those who did not or rarely engaged with health-management practices indicated an interest in doing so in the future, several indicated a need for support or training to be able to manage existing digital resources. These findings highlight a key concern for CALD women, who are known to have lower health literacy levels than women in general [15,46,47].

A US study of pregnant women’s use of information and communication technology by race and ethnicity (“white women,” 23%; Latina women, 28%; African American women, 40%; and women of other ethnic groups, 9%) found that the vast majority owned mobile devices; however, those who had limited English proficiency (24%) and had not graduated from high school (24%), predominantly found in the Latina women group, showed lower usage patterns [48]. In addition, Latina women reported lower use of smartphones (55%), social networking sites (55%), and the internet (62%) than white or African American women; they also sought Web-based health information less (51%). The authors concluded that the uptake of Web-based apps was lower among low-income women, a group overrepresented in Latina and African American women, and that low English language proficiency and literacy were strong barriers to accessing the internet. They recommended that in these groups, alternatives such as paper or translations should be made available and interventions should be developed incorporating culturally and linguistically appropriate elements designed for people with low literacy in their first and second languages [48]. A recent New Zealand study on a short message service (SMS) text message-based maternal health information program reiterates these recommendations. In this study, the uptake and acceptability of a linguistically and culturally tailored program for Maori, Pacific, Asian, and South Asian families was high, and findings confirmed that women had greater access to information and felt supported by the regular messages they received [49].

Finally, previous studies focusing on CALD groups have recommended modifying existing health care procedures to facilitate service access for CALD pregnant women [50-52]. It is likely that the same recommendations should apply for pregnancy apps. CALD women have described struggling with incompatibility between the Western biomedical approaches to maternity practices and those of their own culture. Stapleton et al found that participants in their study were concerned that “the norms of the Australian maternity culture devalued, or indeed disregarded the intergenerational knowledge they viewed as precious, and which they understood offered the best protection for themselves and their infants” [11], a finding that is mirrored elsewhere [53]. The only study identified investigating CALD usage of pregnancy apps [30] advocated pregnant women’s involvement in the app design to increase the effectiveness and usability and incorporated culturally sensitive components such as translations into CALD patients’ languages, pictures of CALD women, and food items familiar to CALD users. During the design phase, participants indicated comprehension difficulties with the app content, which led the researchers to revise the language to make it more understandable. This app was yet to be evaluated, but initial prototype testing indicated participants’ acceptance of the resource.

In this section, we have examined studies that present varying results regarding the uptake of digital resources by CALD women and also regarding the appropriateness or usefulness of these resources for CALD groups. It is possible that the extent to which language and culture create barriers for individual CALD groups or people accounts for these divergent results. It seems reasonable that studies excluding non-English speakers will encounter fewer barriers to resource uptake than those with a representative proportion of participants with no or low English proficiency. This idea warrants further examination. Furthermore, it makes sense that CALD groups will be more inclined to accept and understand digital resources designed in consultation with them and using culturally sensitive methods.

#### Areas for Concern: Regulation and Validity and Reliability of Content

Previous studies have brought attention to the poor quality of some health-related apps [54,55] and even their potential to encourage harmful behaviors [56]. Although pregnancy apps offer many potential benefits, there are problems associated with their development and testing prior to release also. Researchers and health professionals have voiced concerns about the lack of regulation in the industry [1,57], as well as privacy and security issues [2,8,58]. In addition, pregnancy apps may provide inaccurate information [59], not include information that women are seeking [44], or have poor functionality [58]. Two separate studies examining the usefulness of pregnancy apps found that <6% (3.3% and 5.5%, respectively) were considered potentially useful by health care providers [1,59]. In a US study comparing 2 nationally endorsed apps [60], <20% of content explicitly addressed the recommended prenatal care content. In addition, examples of incomplete or confusing information were found, and the researchers reported significant gaps in the educational content, for example, postpartum contraception planning information was omitted, despite its inclusion in the American College of Obstetrics and Gynecology’s guidelines for prenatal care.

Literature examining the perspectives of health professionals highlights other potential issues. A key concern for health professionals is the use of apps or other forms of eHealth as a replacement for members of the maternity health care team [24,61]. A number of other risks were identified by health professionals in qualitative studies of pregnancy mobile health interventions that included potential harm to “the personal or professional integrity of health professionals and health organizations (intellectual property, privacy, and legitimacy concerns),” the danger for misinterpretation if information was taken “out of context,” and an undermining of professional legitimacy or control as women increase their reliance on health-related technology over health professionals “on the ground” [24]. In another study, challenges to professional legitimacy were a concern for health professionals in settings where professional and personal boundaries may become blurred, such as social media forums [62]. Finally, there is evidence that a self-perceived lack of technical skills can impinge on health professionals’ willingness to engage with new technologies as a part of maternity care [62].

Patients may also be wary of the reliability of pregnancy apps or other eHealth content and have demonstrated concerns about personal data security [22,63]. A 2013 Australian study found that while the internet was the first-used source of information by most pregnant women, it was the least trusted [17]. In contrast, health care professionals and hospital print material were the most trusted sources. These are important concerns, particularly among certain groups of pregnant women. Pregnant adolescents, a high-risk group that is less likely to seek health professional advice and care, have been found to prefer apps as their information source for pregnancy education [64].

A lack of health professional guidance for women in their information seeking is an additional concern. Younger women with less experience of pregnancy [19] and first-time mothers [57] tend to use apps more but are particularly vulnerable to noncredible information sources as they are often active information seekers but less likely to know what to expect in pregnancy and childbirth. A German study found a statistically significant association between smartphone app use and younger maternal age, first pregnancy, lower self-rated health, and “influenceability” [65]. Another study found that young first-time mothers exhibited increased levels of anxiety and tension as a result of their information seeking [57]. Other studies have described how women’s Web-based researching can turn into a stressful exercise where women may find it difficult to know when to stop searching [21], where unnecessary worry can be created from exposure to “horror stories” and “scare mongering” [66] and the net result can be more questions or confusion rather than answers [29]. These findings highlight a need for health professionals to have high levels of awareness of available information and resources, which has been demonstrated not to be the case in previous studies [62].

#### A Need for Expert, Reliable Advice From Local Sources

Pregnant women have expressed a need to be able to ascertain the credibility of information in apps. In one Australian study, some specified wanting “expert, credible, up-to-date advice,” and others noted that they would like more “Australian-specific or locally based information” [8]. A 2013 Australian study found that women wanted “clinically endorsed” apps linked to trustworthy websites [23]. Another study found that women want apps and other digital media to give them “ready and instantaneous” access to expert professional information via real-time services such as Web-based messaging or video programs, for example, Skype [6]. The highest response rate for perceived weaknesses in pregnancy apps by respondents in a Korean study [19] was the “lack of credibility” (39%). Almost half of the participants (45%) expressed a need for expert opinions and opportunities for question-and-answer sessions on diet and medication administration during pregnancy. In the same study, the app evaluation component determined that disclosure of information sources in pregnancy apps scored lowest (“lower than average”) on a credibility scale, signally the potentially low trustworthiness of pregnancy app information in general.

Currently, most app developers are commercial entities or internet portals [19]. Health professional and institution involvement in the creation or endorsement of digital information sources is very low [20], although improving [58]. In this scenario, the user becomes a commodity with marketable data, which can be sold to data-mining companies [7], raising concerns about the use, governance, and confidentiality of such data. Pregnancy app users are aware of this and indicate that given the choice, they would prefer apps that are “not linked to the manufacturers of pregnancy or baby products” [8].

As well as calls for the creation of a regulatory framework [2], researchers have recommended that health professionals and health institutions take the initiative to provide both reliable information on maternal health through such mechanisms as apps, or advice about the reliability or validity of other available electronic information [2,57], a desire echoed by patients [23,24]. Health professional engagement with popular modes of information delivery is essential to ensure health services for women are responsive to the changes in information-seeking behaviors and attract rather than alienate women [61]. One reason women use digital media to find information is that they are not able to spend sufficient time with health professionals to have all their questions answered. It is, therefore, imperative that pregnant women can be confident that the information they are accessing is accurate. Johnson [67] has presented a compelling argument that pregnancy apps are effectively performative devices, exerting a strong influence on conceptions and enactments of motherhood in today’s society, and the encouraging the “responsibilization” of health among users. Taking this perspective, the important role of health professionals and health institutions in the design and provision of pregnancy apps is clear and shifts the primary agenda from material gain for commercial entities to evidence-based, expert-endorsed health education for pregnant women.

## Discussion

This review indicates that pregnancy apps are here to stay and confirms that women are increasingly using apps when seeking pregnancy-related information. The findings suggest that to ensure the uptake from women, future pregnancy app resources will need to include not only health services-endorsed information but also incorporate the Web-based and personalizable features that appeal to women currently using pregnancy apps. We have found substantial gaps in multilingual digital resources for the CALD population in Australia and elsewhere, as well as evidence of decreased uptake because of widespread lower literacy and health literacy status of this and other hard-to-reach populations. One of the very appealing aspects of digital and especially mobile technologies is to improve patient care for “unreachable populations” by overcoming the limitations imposed by cost and access [58]. However, this review has shown that there is conjecture regarding the extent to which such marginalized populations benefit from digital resources.

The provision of digital technologies such as apps is fast becoming standard practice in health services, but steps must be taken to ensure that these resources are fit for purpose. The studies discussed in this review have identified that addressing literacy and language barriers may be key components in ensuring the uptake of digital technologies such as apps among CALD and possibly other disadvantaged groups. Research exploring the relative effectiveness of different means of communicating information through the type of multimedia interface that an app offers, for example, graphs, pictures, audio, video, SMS text message lists, and more or less detailed textual information, is recommended to address knowledge gaps in this area. Consideration should be given to include different types of users, including those with higher or lower education levels and literacy, as well as to address cultural, social, and educational barriers to effective use of such resources. Addressing cultural components will be crucial to enhance the appeal and usability of resources for CALD women and will require sensitivity and awareness to ensure appropriateness. We note that one review of currently available apps comments on the ubiquity of white-skinned women and white or pink babies in depictions of pregnant women and their newborn infants [7], a feature that will need modification for such pregnancy apps to be culturally inclusive.

This review has highlighted the lack of reliable information provided by a high proportion of currently available apps—both in terms of content and functionality—as well as a clear demand for trustworthy, locally based, and professionally endorsed pregnancy information resources. It is essential to be able to ensure that information received by high-risk groups, such as adolescent and first-time mothers, is accurate and appropriate for their needs. The possible repercussions of not acting to ensure that the app content is reliable are potentially serious. This problem can be addressed through greater health professional engagement with and oversight of pregnancy app resources from a design perspective. It is important to mention that determining what information qualifies for inclusion in pregnancy information resources is not a straightforward matter. There are diverse views about how much information women should be given about pregnancy-related problems, for example, risks that may or may not become eventualities in individual cases [68]. This is an aspect of pregnancy app design that will require extensive consideration and necessitates health professional input, if not consensus.

In addition, it is suggested that health professionals guide women in their use of apps during pregnancy and that professionally endorsed apps be used adjunctively by health professionals as part of their maternity health care protocol. To ensure the accessibility and uptake of reliable app-based pregnancy information, the provision of free hospital-endorsed apps with complementary resources, such as supportive training and education to use them [16], is also recommended. To help achieve these objectives, more research into the effectiveness of apps is also required. Consideration of the issues raised in this review has markedly contributed to a pregnancy app resource developed by the authors [69], and it is hoped that similar consideration is given to the development, design, and testing of any pregnancy-related app resource in the future.

## Appendix

Multimedia Appendix 1 Sample search strategy (MEDLINE search for type 1 studies).

Multimedia Appendix 2 Papers examining women’s use and preferred features in pregnancy apps, and issues in pregnancy apps use.

Multimedia Appendix 3 Papers including culturally and linguistically diverse women’s use of digital technology during pregnancy.

## Abbreviations

CALD: culturally and linguistically diverse
eHealth: electronic health
NESB: non-English-speaking background
SMS: short message service
