# Supplementary figures and images for: The Rise of Pregnancy Apps and the Implications for Culturally and Linguistically Diverse Women: Narrative Review
Source: JMIR Mhealth Uhealth. 2018 Nov 16;6(11):e189. doi: 10.2196/mhealth.9119 (PMC6269626; doi:10.2196/mhealth.9119)

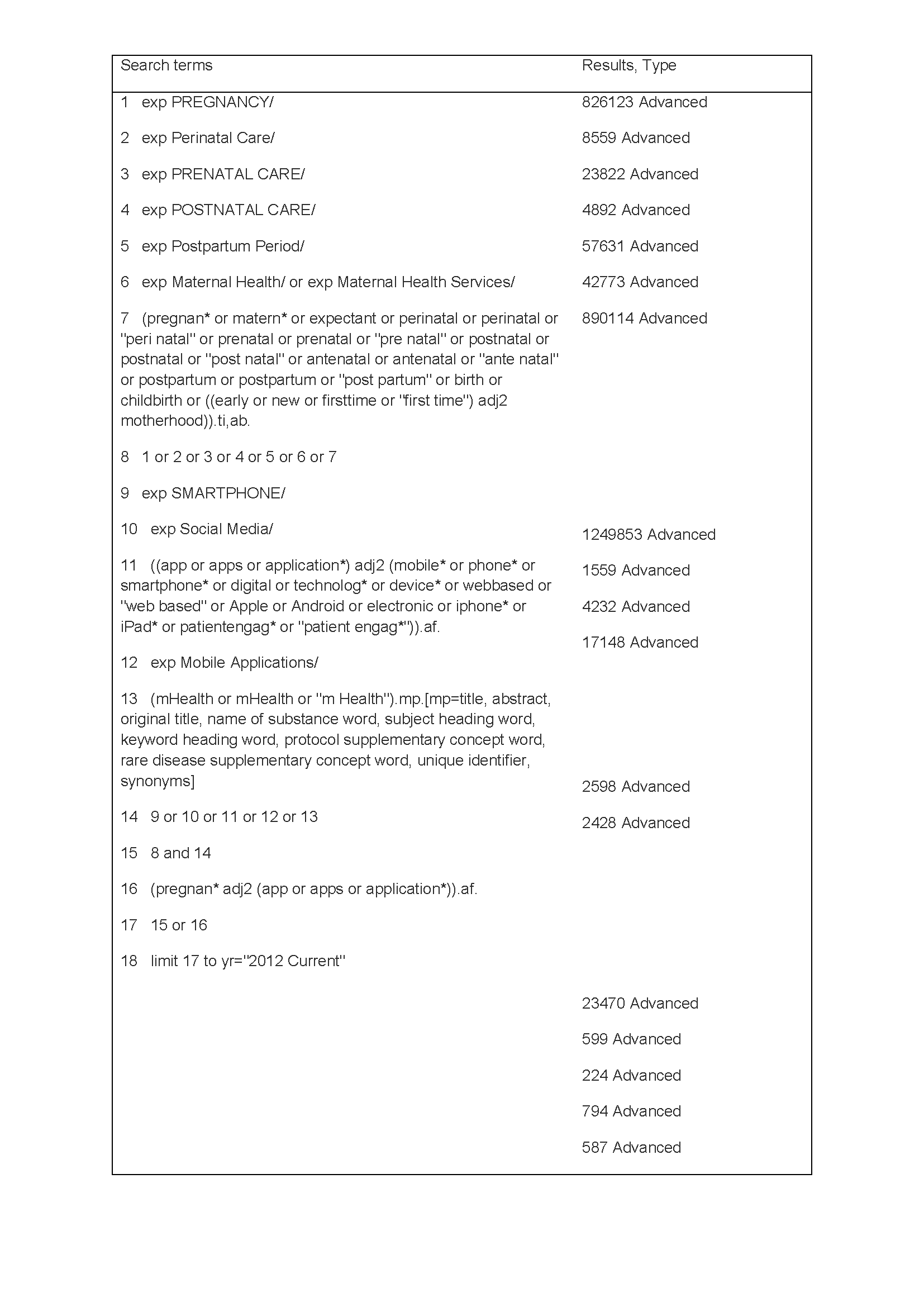

Supplement: Multimedia Appendix 1 [file mhealth_v6i11e189_app1.png]
