# Supplementary material for: The Rise of Pregnancy Apps and the Implications for Culturally and Linguistically Diverse Women: Narrative Review
Source: JMIR Mhealth Uhealth. 2018 Nov 16;6(11):e189. doi: 10.2196/mhealth.9119 (PMC6269626; doi:10.2196/mhealth.9119)
Supplement: Multimedia Appendix 2 [file mhealth_v6i11e189_app2.pdf]

Article	Research method	participants/ data source	Ethnicity, socioeconomic and educational levels of participants (where applicable)	Participants who used apps	Preferred functionalities/ positive features	Identified issues with using apps	Location
Bert et al (2015)	app review	The 9 most commonly cited pregnancy-related apps	N/A	N/A	• All apps were available from the Apple App Store, almost all were available from the Google Play Store.<br>• Most apps were entirely free, or at least some functionality was free.<br>• These apps provide information on "all the different aspects of the gestation..."<br>• Social connection - direct links to forums	• Only two apps were available in multiple languages.<br>• 6/9 apps did not include information about the reliability of app content and/or the presence of a scientific board ensuring evidence-based contents.<br>• 4/9 apps did not contain a privacy policy.	International
Dalton et al (2014)	focus groups, interviews and survey	21 midwives	N/A	N/A	• May help to alleviate time constraints as a factor in communicating information to women during appointments<br>• One-to-one communication rather than group	• Communication with women in online contexts is problematic because of difficulties in correctly interpreting "tone", lack of non-verbal cues and ability to observe women's condition physically; medicolegal risks for health professionals;<br>• the potential and danger for misinterpretation of information given to be taken “out of context”;<br>• the potential blurring of professional and personal roles<br>• Moving to online information provision may result in added work<br>• Uncertainty about the quality of information being provided.	Adelaide, Australia
Farag, Chyjek & Chen (2014)	app review	548 pregnancy apps in the Apple App Store	N/A	N/A		30/548 (5.5%) apps were considered "potentially useful to obstetricians-gynaecologists"	international

Article	Research method	participants/ data source	Ethnicity, socioeconomic and educational levels of participants (where applicable)	Participants who used apps	Preferred functionalities/ positive features	Identified issues with using apps	Location
Fleming, Vandermause & Shaw (2014),	Focus groups	7 primiparous women who had recently given birth<br>Diverse maternal care providers (12 across 3 focus groups)<br>app investigation	low-income women	not stated		• Pregnant women's information-seeking can result in increased anxiety and fear.<br>• Most women did not talk to their health care providers about their information-seeking.<br>• Information acquired from electronic sources was often "fragmented, inconsistent, ... poorly referenced"<br>• Women often received advice from new "friends" in online communities<br>• Few of the pregnancy or birthing apps included contact information or 'about us' sections.	Pacific North-West, U.S.
Goetz et al (2017)	interview and questionnaire	30 pregnant women	highly educated, high socioeconomic status	60% were "regular users"	• interactive/pregnancy tracking<br>• accessibility/convenience<br>• social connection<br>• reliable source/link to clinical care<br>• data sharing	trustworthiness of content<br>data security	Germany
Hearn, Miller & Fletcher (2013)<br>Hearn, Miller & Lester (2014)	Interviews and focus groups<br>App and website launch	76 health care providers<br>56 pregnant women<br>64 postnatal women	urban and rural participants, low and medium socioeconomic levels	7% of all pregnant women and 18% of first-time mothers used the app in its first year	Women:<br>• reliable source/link to clinical care<br>• succinct information provision (with links to more detailed information if wanted)<br>• Interactive/pregnancy tracking<br>• social connection<br>• information provision in stages of pregnancy format<br>Health care providers:<br>• information provision<br>• links to support<br>• easy and fun interface (include images, animations, etc)<br>• interactive	• trustworthiness of content lower use in areas of social disadvantage.<br>• specialised content needed for indigenous and non-English speaking women.<br>• possible internet/smartphone access issues in remote/ disadvantaged areas.	Western Australia, Australia
Johnson (2014)	Interviews	12 perinatal women	No information provided	N/A	• accessibility/convenience<br>• succinct information provision - 'tidbitisation'		Sydney, Australia

Article	Research method	participants/ data source	Ethnicity, socioeconomic and educational levels of participants (where applicable)	Participants who used apps	Preferred functionalities/ positive features	Identified issues with using apps	Location
					• social connection		
Kennedy et al (2017)	questionnaire	101 pregnant women	No information on ethnicity given,<br>Range of educational and socioeconomic levels represented	87%	• Social connection<br>• Information provision<br>• Accessibility/convenience<br>• interactive/pregnancy tracking	Women used mainly commercially developed resources, not publicly-funded or academically-supported resources, raising the possibility of them receiving conflicting or erroneous advice.	Dublin, Ireland
Kraschnewski et al. (2014) Peyton (2014), C	Focus groups only (Kraschnewski), Focus groups and interviews (Peyton)	17 pregnant women (4 focus groups), 6 perinatal women (interviews), all smartphone owners	"Mostly white" (14/17 in focus groups),<br>lower socioeconomic status	100%	• information provision<br>• Interactive/pregnancy tracking<br>• social connection<br>• Regular notifications/reminders<br>• Interactive (eg. photo sharing capability, personalisation)<br>• Succinct information provision<br>• Stages of pregnancy format/pregnancy tracking	• trustworthiness of content<br>• accessibility concerns (ehealth literacy); Lack of support in apps for spouses. Gendered design of apps is alienating for spouses.<br>• Disinterest in and distrust of pregnancy discussion forums, social media venues, and chat rooms.<br>• Too much information seeking can lead to confusion	Pennsylvania, US
Lee & Moon (2016)	user survey and app review	193 pregnant women (smartphone owners)<br>The apps (n=47) used by the women	The majority of participants had at least an undergraduate degree (73.1%). All had completed high school.<br>Most were of medium (38.3%) to high (46.1) socioeconomic status.	55%	• information provision<br>• accessibility/convenience<br>• Social connection<br>• link to clinical care (Q&A sessions)	Women:<br>• trustworthiness of content<br>App review:<br>• provision of information sources and warnings about using information related to pregnancy, birth and child care had low credibility<br>• internet portal or commercial sites were the most common developers of apps, not healthcare organisations.	Daegu, Korea
Lupton & Pedersen (2015)<br>Lupton & Pedersen (2016)	Survey	410 pregnant or recently pregnant women	Predominantly city dwellers, highly educated, Anglo or Western European background	73%	• information provision<br>• Interactive/pregnancy tracking<br>• social connection		Australia-wide
Lupton (2016)	Focus group (follow up to	36 pregnant or recently pregnant women (4 focus groups)	English-speaking, city dwellers, highly-educated	High	• Multifunctionality<br>• accessibility/convenience<br>• social connection<br>• reliable source/link to clinical	agendas of commercial entities	Sydney, Australia

Article	Research method	participants/ data source	Ethnicity, socioeconomic and educational levels of participants (where applicable)	Participants who used apps	Preferred functionalities/ positive features	Identified issues with using apps	Location
	quantitative survey)				care		
O'Donnell, Lewkowitz, Vargas & Zlatnik (2016)	qualitative app analysis	Two free, nationally-endorsed (U.S.) pregnancy apps	N/A	N/A		• Content between apps varied widely; one had no information on: alternative medications and exercises, constraints to physical acitivity, labour and delivery and postnatal care<br>• In both apps, less than 20% of content explicitly addressed recommended prenatal care information<br>• Examples found of incomplete/confusing information<br>• Gap in educational content (no information regarding postpartum contraception planning)	U.S.
O'Higgins et al 2014	Questionnaire	522 perinatal women	literate (English language), urban and rural participants, spanning all socioeconomic groups<br>No information regarding ethnicity	Of smartphone owners (76%), 59% had used a pregnancy app	• Regular notifications/reminders<br>• Information provision in stages of pregnancy format (eg. weekly texts)<br>• Videos<br>• Specific apps (general pregnancy, nutrition, exercise, smoking cessation)<br>• Social connection (forums, blog)		Dublin, Ireland
Prescott & Mackie (2017)	Interview	16 pregnant women	No information provided	not stated	• reliable source<br>• information provision<br>• Interactive/pregnancy tracking	• trustworthiness of content<br>• difficulty knowing when to stop looking<br>• access to too much information ("horror stories")	England
Rodger et al. (2013)	interview	35 pregnant women	predominantly Anglo-Australian (32/35), and from a low socioeconomic area (66%)	40%	accessibility/convenience	trustworthiness of content (anecdotal)	South Australia, Australia

Article	Research method	participants/ data source	Ethnicity, socioeconomic and educational levels of participants (where applicable)	Participants who used apps	Preferred functionalities/ positive features	Identified issues with using apps	Location
Scott, Gomeichards & Caldwell (2014)	app review	Top 10 free maternal and child health apps (in the Google Play and Apple App Stores)	N/A	N/A		• Only 2 apps were assessed to meet functional requirements, security, health professional involvement and evidence-based medical content (but they were not fully usable)<br>• 4/10 apps were fully functional<br>• 3/10 apps had adequate security mechanisms for data privacy<br>• 2/10 apps were fully usable<br>• 4/10 involved health professionals in their development and evaluation.<br>• 4/10 apps provided information from evidence-based medical sources.	International
Tripp et al (2014)	app survey	1049 apps iTunes, 497 apps Google Play	N/A	N/A	• Interactive/pregnancy tracking<br>• Information provision		International
Wallweiner et al (2016)	questionnaire	220 pregnant women	Women had to have sufficient knowledge of German to participate<br>A range of education levels represented, majority with university entrance qualification or higher	22%		Women who used apps tended to be younger, in their first pregnancy rated themselves as less healthy and were the most influenced (of the participant group) by the information they received, raising concerns about potential outcomes given the known unreliability of mobile application content.	Heidelberg and Tubingen, Germany
Wierckx, Shahid & Al Mahmud (2014)	Interviews and prototype evaluation	Interviews:<br>13 nulliparous pregnant women<br>3 obstetricians<br>Evaluation:<br>11 nulliparous pregnant women<br>1 obstetrician	No information provided	N/A	Women:<br>• Information provision<br>• accessibility/convenience<br>• Interactive/pregnancy tracking<br>• Social connection<br>• Communication platform between women and health professional<br>• Integration with clinical pregnancy care<br>Obstetricians:<br>• information provision	Obstetricians:<br>Reluctance to be contactable via an app (increase in workload)	Netherlands

Article	Research method	participants/ data source	Ethnicity, socioeconomic and educational levels of participants (where applicable)	Participants who used apps	Preferred functionalities/ positive features	Identified issues with using apps	Location
Willcox et al (2015)	Focus groups and interviews	15 pregnant or postpartum women<br>12 health professionals	Women: two were born outside Australia. No other information given<br>Health professionals: two each of obstetricians, general practitioners, midwives, dieticians, physiotherapists, community pharmacists	93.3% (14/15)	Health professionals:<br>• Accessibility<br>• Adjunct to health professional care<br>• reliable source<br>Women:<br>• Accessibility<br>• Reliable source/Link to clinical care<br>• Videos<br>• Social connection<br>• Personalisation<br>• Concise information, delivered in intervals<br>• Integration of technology capability (cross-platform)	Health professionals:<br>• access problems for disadvantaged women<br>• medicolegal risks for health professionals<br>• the potential and danger for misinterpretation of information taken "out of context"<br>• increased reliance on health-related technology over health professionals<br>• lack of regulatory body and health funding for technology<br>Women:<br>• fear of unsupportive peers or privacy breaches on social networks or forums<br>• underutilised apps they already had	Melbourne, Australia
