# Supplementary material for: The Rise of Pregnancy Apps and the Implications for Culturally and Linguistically Diverse Women: Narrative Review
Source: JMIR Mhealth Uhealth. 2018 Nov 16;6(11):e189. doi: 10.2196/mhealth.9119 (PMC6269626; doi:10.2196/mhealth.9119)
Supplement: Multimedia Appendix 3 [file mhealth_v6i11e189_app3.pdf]

Article	Research method	Participants/data source	Ethnicity, socioeconomic and educational levels of participants (where available)	Relevant findings	Recommendations	Location
Bensley et al (2014)	Online survey (available in English and Spanish)	8,114 women or caregivers of young children.	Hispanic (47%) or Other (53%) comprising: White (57%), African American (7%), American Indian/Alaskan native (7%), Asian (6%), Native Hawaiian/Pacific Islander (3%), other or missing (20%)	• The majority (92%) owned a cell phone, (95% of millennials), over half (58%) were smart phones.<br>• Only 31% of respondents with smart phones used smart phone apps for parenting and health-related information.<br>• Younger respondents (< 25yrs) were more likely to use a cell phone than another device to access the internet.<br>• High interest in receiving nutritional information, accessing support groups and services via technology in the future	Technologies to consider for improving govt. nutritional program service provision:<br>• text messaging for appointment reminders and nutrition education,<br>• online scheduling and nutrition education,<br>• smart phone applications to locate nearby service offices and vendors, to access approved food lists, and scan foods at the store<br>• a stronger Facebook presence for interacting with clients and breastfeeding support.	Western USA
Brusk & Bensley (2016)	Data analytics on website usage	305,735 unique clients of a US-govt nutritional education program who completed 612,201 lessons over a 1-year period.	White, Black, Hispanic, Other/missing (proportions not given).	• In an evaluation of access methods for online nutritional education, Spanish language and black race (as well as residing in Alabama) were associated with a higher likelihood that the user completed their lesson using a mobile device.<br>• Low user engagement with program lessons was correlated with accessing the content via a mobile device	Future research could address:<br>• how the type of device used to access content affects user engagement<br>• whether mobile device Internet access was the initial means for these users to gain access to the Internet on a regular basis<br>• Design and functionality needs to cater for mobile device users	US (across 21 states)

Article	Research method	Participants/data source	Ethnicity, socioeconomic and educational levels of participants (where available)	Relevant findings	Recommendations	Location
Carolan-Olah, Steele & Krenzin (2015)	Pre- and posttest questionnaire to assess web-based intervention	21 pregnant women with gestational diabetes mellitus	"conversational English"<br>Country of birth: Vietnamese (6), Australian (5), India (5), China (3), Phillipines (1), Sudan (1)<br>Educational level: did not complete high school (11), completed high school (9), no answer (1)	• Almost all participants found the intervention acceptable and pertinent to their information needs Almost all participants found it at least fairly easy to use and fairly useful.<br>• 70.6% of participants improved their knowledge scores post-intervention 50% improved their knowledge of food score<br>• 36.8% improved their self-management score	From participants:<br>• larger pictures (10/21)<br>• more ethnic specific foods (11/21)<br>• less ethnic specific foods (7/21)<br>• less text (3/21)<br>• more information (6/21), including recipes suitable for GDM.	Melbourne, Australia
Chilukuri et al (2015)	Survey	246 pregnant women and mothers of children < 1 year old	Literate (in English or Spanish)<br>28% Latina, 40% black, 23% white, 9% other racial/ethnic groups	• 95% of participants were mobile phone users<br>• 74% smartphone users (55% Latinas, 77% African Americans, 84% whites, 78% other races)<br>• 85% internet users (62% Latinas, 92% African Americans, 96% whites, 96% other races)<br>• 74% used the internet to find health information (51% Latinas, 79% African Americans, 88% whites, 91% other races)<br>• low English language proficiency and literacy are strong barriers to accessing the internet	• provide alternatives (paper) or translations<br>• development of interventions incorporating culturally and linguistically appropriate elements, and designed for people with low literacy	Baltimore, Maryland, US
Dobson et al (2017)	Evaluation of multilingual texting program using: program data analytics phone interviews	Pregnant women and mothers of young children, and other family members: data from 1,404 individuals collected from content management system for a texting program 29 interviews	Maori, Pacific Islander, Chinese, Korean, Japanese and other Asian	Engagement:<br>• 1,404 individuals enrolled in program in first 18 months<br>Acceptability:<br>• 93% found messages useful,<br>• 97% found them relevant,<br>• 100% found messages culturally appropriate and easy to understand,<br>• 50% reported behavioural improvements from the program,<br>• 100% felt supported by program<br>• 72% reported increased knowledge	Bidirectional messages would be useful.	Two urban districts in New Zealand

Article	Research method	Participants/data source	Ethnicity, socioeconomic and educational levels of participants (where available)	Relevant findings	Recommendations	Location
				and understanding.<br>• Participants also liked the personalisation of messages.		
Garnweidner-Holme et al (2015)	Interviews with women (user-involvement design), health professional/ researcher group discussions	21 pregnant women with GDM, researchers, health professionals	Norwegian (10), African (2), Asian (5), Eastern European (2), South American (2)	During first interview (with app prototype):<br>• women had difficulties understanding some app content and medical terminology<br>• Some women were confused about information that conflicted with health professional advice they had received<br>During second interview (speak-aloud protocol, updated app)<br>• Only 9 participants were frequent users of apps but all (n = 21) stated that they would use the app – which featured culturally sensitive components such as Urdu and Somali translations, pictures of women from various ethnic backgrounds, and food items familiar to the cultures in question.<br>• Participants performed very well overall on six prescribed tasks<br>Women liked the following features:<br>• Personalisation<br>• Interactive (real-time blood sugar level readings)	• Involve patients and health professionals directly in the design of apps to increase effectiveness and usability<br>• Ensure close collaboration between app developers and healthcare professionals to eliminate potential confusion.	Norway
Grimes, Forster & Newton (2014)	Postal questionnaire	350 primiparous women who had given birth in previous 4 months	All socioeconomic and educational levels represented<br>118 participants were born outside Australia, 122 identified as NESB	internet cited as the most used source of pregnancy information by NESB women		Melbourne, Australia

Article	Research method	Participants/data source	Ethnicity, socioeconomic and educational levels of participants (where available)	Relevant findings	Recommendations	Location
Guendelman et al (2017)	Focus groups	92 pregnant women and mothers of young children (14 focus groups)	literate (in English)<br>low socioeconomic status<br>8 white, 40 black, 22 Latina, 15 Asian, 7 mixed race or other race	• very high access to smartphones and computers<br>• high use of the Internet for health information-seeking<br>• low use of digital tools for health management practices		U.S.-multi-site
Guerra-Reyes, Christie, Prabakar & Siek (2017)	survey	77 postpartum women	American Indian or Alaska native (1.3%), Asian (6.5%), Black or African-American (5.2%), White (74%), More than one race (5.2%, not reported (7.8%), Hispanic or Latino ethnicity (3.9%)<br>Range of education levels, predominantly with some college (28.6%) or with a Bachelor's degree or higher (42.9%). High school or less 20.8%.<br>Low income (42.9%) and high income (57.1%)	• 77% of participants owned smartphones<br>• 48% of those with smartphones used pregnancy apps	Participants reported using varied sources of health information during pregnancy, but 44% still experienced one or more postpartum gaps in health information.	Monroe County, Indiana, U.S.
Narasimhulu et al (2016)	survey<br>expert review of online information	503 pregnant/postpartum women<br>5 experts	Able to read and write in English (although, primary language was other than English for 38.1% of participants)<br>An underservedacially diverse inner-city population: White (38.9%), African American (16.4%), Hispanic (13.4%), Asian (25.7%), other (5.6%)	Women<br>• 70.8 % were e-health users.<br>• E-health users were younger, more likely to be nulliparous, have English as their primary language and have a college education.<br>• 60 % said e-health influenced their decision making<br>• Only 71.3 % discussed their searches with their provider.<br>• Device most commonly used to access the internet: 85% computer, 67.1% smartphone, 34.4% tablet, 0.7% other.<br>Expert review of pregnancy information websites:<br>• For each of five pregnancy questions, at least one website out of ten	Healthcare providers have a vital role in suggesting high-quality online information.	Brooklyn, NY, US

Article	Research method	Participants/data source	Ethnicity, socioeconomic and educational levels of participants (where available)	Relevant findings	Recommendations	Location
				reviewed was found to provide inaccurate information.		
Osma et al. (2016)	online survey	249 English-speaking and 247 Spanish-speaking pregnant or postnatal women (from 59 countries)	African-American/Black (8.2%), Alaskan native or American Indian (0.7%), Asian (18.4%), European American or White (21.5%), Mestizo (24.4%), Native Hawaiian (0.4%), Pacific Islander (0.7%), More than one race (3.1%), others (22.6%)<br>Middle (85.5%) and Low (14.5%) socioeconomic status,<br>Range of education levels but highest group university level (39.9%)		• 47.5% of sample owned mobile phone (but of these, 72.3% used health-related apps).<br>• Significant differences in socioeconomic status were found for Internet seeking behaviour of health-related information and downloading apps between those with and without Internet access (presumably because the latter don't own ICT devices - in spite of the fact that the survey was completed online).	international
Peragallo Urrutia et al. (2015)	Written survey	100 pregnant women	61 white, 26 black, 6 Hispanic and 7 Asian women	• Very high general use of and access to the Internet via computer (89%) and/or mobile phone (89%), slightly less access amongst non-white women and those with children already.<br>• 49% were willing to participate in a mobile-based internet intervention, 83% in a computer-based intervention.<br>• No statistically significant differences for race or income level		southern U.S.
Sundstrom (2016)	qualitative interviews	44 postnatal women	English-speaking,<br>White (64%), Hispanic (25%), Black (7%), Asian (4%)<br>Education level less than college (56%), College degree or higher (44%)	• A high proportion of respondents used pregnancy apps (number not stated).<br>• Reliable source<br>• Social connection (but not as contributors, only observers)	Some participants chose not to use apps during pregnancy due to privacy concerns	Urban U.S.

Article	Research method	Participants/data source	Ethnicity, socioeconomic and educational levels of participants (where available)	Relevant findings	Recommendations	Location
Waring et al (2014)	questionnaire	64 pregnant women	English speaking Non-Hispanic White (48%), African American (13%), Hispanic (28%), other race/ethnicity (11%) Education level high school or less (42%), high school or some college (30%), college degree or higher (28%)	• 94% of participants looked online for pregnancy information<br>• 95% had used the Internet in the past four weeks (89% daily)<br>• Smartphones were the most commonly used device (84% of participants), followed by 66% via laptop, 49% via desktop computer, 31% via tablet, and 23% another device.<br>• 86% overall were interested in using a website or mobile application to help them gain a healthy amount of weight during pregnancy	The study results suggest that Internet-delivered gestational weight gain interventions should be designed to be accessible via smartphones and other mobile devices in addition to via desktop or laptop computers.	US
